# Timing of physical activity across adulthood on later-life cognition: 30 years follow-up in the 1946 British birth cohort

**DOI:** 10.1136/jnnp-2022-329955

**Published:** 2023-02-21

**Authors:** Sarah-Naomi James, Yu-Jie Chiou, Nasri Fatih, Louisa P Needham, Jonathan M Schott, Marcus Richards

**Affiliations:** 1 MRC Unit for Lifelong Health and Ageing at UCL, University College London, London, UK; 2 Department of Psychiatry, Chang Gung Memorial Hospital Kaohsiung Branch, Kaohsiung, Taiwan; 3 Nuffield Department of Population Health, University of Oxford, Oxford, UK; 4 Dementia Research Centre, UCL Queen Square Institute of Neurology, London, UK

**Keywords:** dementia, cognition, neuroepidemiology, alzheimer's disease

## Abstract

**Background:**

To assess how timing, frequency and maintenance of being physically active, spanning over 30 years in adulthood, is associated with later-life cognitive function.

**Methods:**

Participants (n=1417, 53% female) were from the prospective longitudinal cohort study, 1946 British birth cohort. Participation in leisure time physical activity was reported five times between ages 36 and 69, categorised into: not active (no participation in physical activity/month); moderately active (participated 1–4 times/month); most active (participated 5 or more times/month). Cognition at age 69 was assessed by tests of cognitive state (Addenbrooke’s Cognitive Examination-III), verbal memory (word learning test) and processing speed (visual search speed).

**Results:**

Being physically active, at all assessments in adulthood, was associated with higher cognition at age 69. For cognitive state and verbal memory, the effect sizes were similar across all adult ages, and between those who were moderately and most physically active. The strongest association was between sustained cumulative physical activity and later-life cognitive state, in a dose-response manner. Adjusting for childhood cognition, childhood socioeconomic position and education largely attenuated these associations but results mainly remained significant at the 5% level.

**Conclusions:**

Being physically active at any time in adulthood, and to any extent, is linked with higher later-life cognitive state, but lifelong maintenance of physical activity was most optimal. These relationships were partly explained by childhood cognition and education, but independent of cardiovascular and mental health and APOE-E4, suggestive of the importance of education on the lifelong impacts of physical activity.

**WHAT IS ALREADY KNOWN ON THIS TOPIC**

- Physical activity is one proposed modifiable risk factor, modestly associated with lower risk of all-cause dementia, cognitive decline and later-life cognition. While some studies indicate that physical activity in midlife or later-life is most beneficial for later-life cognition, studies often have short follow-up periods, or are at risk of reverse directionality.
- Midlife is an important period of risk exposure for cardiovascular health on later-life cognition and brain health, yet it is unclear if there are important, so-called ‘sensitive’, periods for physical activity exposure, or an influential role of sustained activity, on later-life cognition.
- Better characterisation of the timing and maintenance of physical activity would help guide interventions to optimise cognitive health in older populations. The relationship between exercise maintenance, referring to the preservation of physical tactivity, and cognitive health outcomes is underexplored.

**WHAT THIS STUDY ADDS**

- We find compelling evidence of an association between being, and remaining, physically active across adulthood on later-life cognition.
- We show that being physically active at any time in adulthood, and to any extent (participating at least once per month), is linked with higher later-life cognitive state; but the strongest link is for those maintaining physical activity for longer, in an accumulative manner.
- Together, these results suggest that the initiation and maintenance of physical activity across adulthood may be more important than the timing of participating in physical activity in the life course, or the frequency of physical activity at a specific period. Lifelong maintenance of physical activity is the most optimal.

**HOW THIS STUDY MIGHT AFFECT RESEARCH, PRACTICE OR POLICY**

- Our findings provide evidence that encouraging inactive adults to be more active at any time across the life span, and encouraging already active adults to maintain activity, could confer benefits on later-life cognition.
- This supports initiatives to encourage participation and maintenance of physical activity at all ages, and for better access to education that develops skills and motivation for physical activity participation.
- Our results warrant further investigation into the pathways underlying inferred benefits of physical activity on later-life cognition.

## Introduction

Dementia currently affects 44 million people worldwide with prevalence expected to triple by 2050.^1^ Potentially modifiable risk factors may confer as much as 40% of dementia risk.^2^ Physical activity is one proposed modifiable risk factor and is modestly associated with lower risk of all-cause dementia,^3 4^ cognitive decline^5^ and later-life cognition.^6 7^ However, it is yet to be established whether the timing, frequency or maintenance of physical activity across the life course confers optimal benefit on later-life cognition. While some studies indicate that physical activity in midlife or later-life is most beneficial for later-life cognition,^6 7^ studies often have short follow-up periods, or are at risk of reverse directionality.^8^ Midlife is an important period of risk exposure for cardiovascular health on later-life cognition and brain health,^4 9 10^ yet it is unclear if there are important, so-called ‘sensitive’, periods for physical activity exposure, or an influential role of sustained and preserved activity throughout life, on later-life cognition. Furthermore, pathways underlying the relationship between physical activity and later-life cognition are not well established. Associations observed could be explained by earlier-life confounders such as education and social class.^11 12^ Implicated pathways conferring causal benefits also include better cardiovascular health^13^ and better mental health.^4^ There may also be a differential effect of physical activity on cognition by genetic APOE ε4 risk status.^14^

Using data from the population-based 1946 British birth cohort, which has followed people born in the same week of 1946, previous studies have demonstrated beneficial effects of midlife physical activity on midlife verbal memory^15^ and search speed^16^ decline. Here, we extend this work by taking a life course approach to evaluate the effects of physical activity timing, frequency and maintenance, spanning over 30 years, with later-life cognitive function. We assess three measures of later-life cognitive function including a measure of cognitive state, verbal memory and processing speed. We further aim to investigate to what extent, these effects are explained by pathways including earlier-life influences, cardiovascular health and mental health.

To investigate the effect of timing of physical activity, we investigated the strength of associations between a range of cognitive tests at age 69 with participation in physical activity at the ages of 36, 43, 53, 60 and 69. We then investigated whether any associations observed are best explained by physical activity in specific ‘sensitive’ periods across the life course, or being physically active across multiple time periods. Finally, we tested whether associations between physical activity and later-life cognition are modified by sex or APOE-ε4; if associations are independent of a range of relevant confounders including childhood cognition, childhood socioeconomic position (SEP) and education; and potential contributory factors including cardiovascular and mental health.

## Methods

### Prospective longitudinal cohort study sample

**Figure 1:**
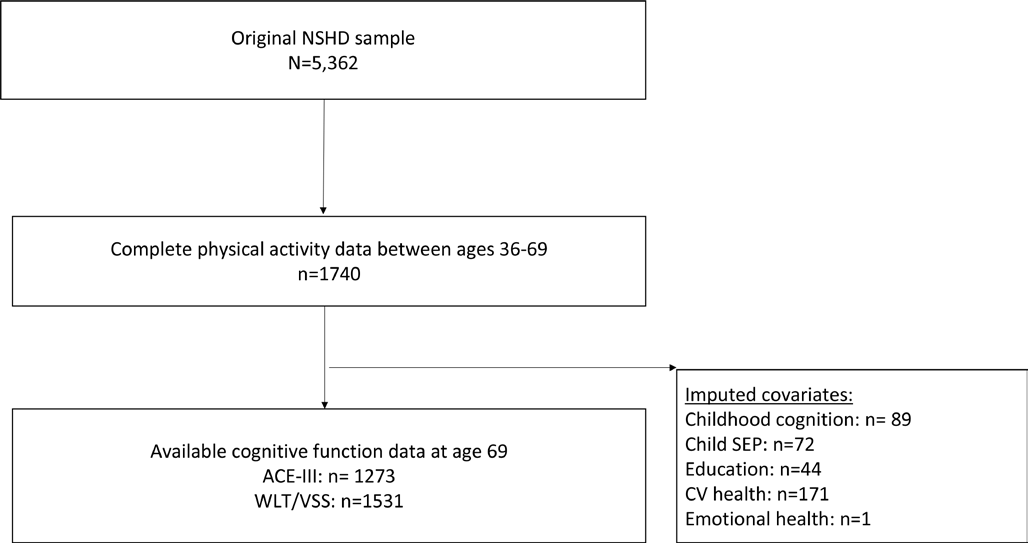
Flow chart of the analytical sample. ACE-III, Addenbrooke’s Cognitive Examination-III; NSHD, National Survey of Health and Development; SEP, socioeconomic position; VSS, Visual Search Speed; WLT, Word Learning test.

Study participants were from the Medical Research Council National Survey of Health and Development (the 1946 British birth cohort) all born in mainland Britain in 1 week of March 1946. Follow-up has included over 24 contacts with the whole sample since birth, with the latest full testing wave conducted at age 69.^17^ Figure 1 outlines the flow chart of the analytical sample.

#### Physical activity

Participation in leisure time physical activity was collected prospectively at ages 36, 43, 53, 60–64 and 69 years. At age 36, participation was ascertained using a modified validated Minnesota leisure time physical activity questionnaire, assessing how often people participated in a range of physical activities per month.^18^ A wider questionnaire was derived partly from the Minnesota leisure time physical activity questionnaire; the EPIC Physical Activity Questionnaire-2. This assessed how often people had taken part in any sports, vigorous leisure activities or exercise in the previous month (version administered at age 43) and the previous 4 weeks (version administered at ages 53, 60 and 69).^19^ Similarly to previous work in the sample, at each age, responses were categorised into: not active (no participation in physical activity/month); moderately active (participated 1–4 times/month); most active (participated 5 or more times/month).^16 20–22^ Previous work in the cohort has demonstrated the consistency and similar patterns of variation between objective and self-reported instruments of physical activity.^23^

To investigate the accumulative effect of physical activity across adulthood, physical activity responses at each age were binarised into: (0) not active (no participation in physical activity/month) and (1) active (participated one or more times/month) and summed across all five time points to create a total score ranging from 0 (inactive at all ages) to 5 (active at all ages).

### Cognitive measures at age 69

At age 69, participants underwent a validated version of the Addenbrooke’s Cognitive Examination-III (ACE-III) administered by iPad using ACEMobile (https://acemobile.frontierftd.org), where this was not possible, a paper version was used (n=10). The ACE-III is a test of cognitive state divided into five domains: attention and orientation, verbal fluency, memory, language, and visuospatial function,^24^ with the total summed score indexing overall cognitive state (max 100). Other cognitive domain tests were administered by study nurses including episodic verbal memory and processing speed. Episodic verbal memory was measured by word learning tests (WLT), assessed by recall of a 15-item word list. The total number of words correctly recalled over three identical trials was summed to provide an overall score for WLT (max 45). Processing speed was assessed by a visual search task, where participants were required to cross out the letters P and W, randomly embedded within a page of other letters, as quickly and accurately as possible, within 1 min. Visual search speed (VSS) represents the total number of letters searched (max 600).^25^

#### Earlier-life covariables

Earlier-life covariables were childhood cognition,^26^ childhood SEP^11^ and education.^27^ The standardised sum of four tests of verbal and non-verbal ability at age 8 represented childhood cognition. Childhood SEP was recorded from paternal occupation according to the Registrar General’s classification of the paternal occupation^28^ and dichotomised into ‘unskilled, partly skilled or manual skilled’ and ‘non-manual skilled, intermediate or professional’. Education up to age 26 was categorised into three groups based on the Burnham Scale^29^: ‘none attempted’, ‘vocational or ordinary (O’ level or equivalent)’ and ‘advanced (A level) or higher education’.

#### Cardiovascular health

A cardiovascular health risk profile at age 69 was inferred from deriving the office-based Framingham Heart Study risk score in line with previous studies,^10 30 31^ using a weighted sum of age, sex, systolic blood pressure (measured after 5 min of rest), history of diabetes (self-reported diagnosis yes/no or haemoglobin A_1c_ level of >6.5%), current antihypertensive medication use (self-reported yes/no), current cigarette smoking status (self-reported yes/no) and body mass index (weight(kg)/height(m^2^)).

#### Emotional mental health

Emotional mental health at age 69 was indexed by the General Health Questionnaire-28 (GHQ-28).^32 33^ In line with a validated threshold, more than four points in a rescaled total GHQ-28 score indicated case-level emotional symptoms.^33 34^

#### APOE-ε4 status

Single-nucleotide polymorphisms rs7412 and rs429358 were examined by KBioscience to ascertain APOE-ε4 genotype. Individuals were categorised as APOE-ε4 carriers or non-carriers.^12^

#### Statistical analysis

The descriptive characteristics are shown as means±SD for continuous variables and proportions for categorical variables. All statistical analyses were performed in STATA V.17 (STATA) with a 0.05 significance level. Participants were included in this analysis if they had complete physical activity data at five assessments and at least one cognitive measure at age 69. Multiple imputation for missing covariate data was performed using the multivariate imputation by chained equation (MICE) method with 50 imputations. MICE results are presented, but complete case data show a similar pattern (see online supplemental eTable 3, online supplemental eFigures 2 and 3).

10.1136/jnnp-2022-329955.supp1 Supplementary data

#### Physical activity with cognition: regression modelling approach

We conducted a series of linear regression models to test the relationship between physical activity measures across adulthood and later-life cognition (total ACE-III, WLT and VSS at age 69 standardised to the analytical sample), adjusting for sex (model 1). Physical activity at each of the five assessment periods was modelled as a categorical variable with ‘not active’ as the reference group. The accumulative physical activity score was modelled as a categorical variable using ‘inactive at all ages’ as the reference group. Tests of linearity of the accumulative physical activity score were further conducted post hoc.

To reduce multiple testing, we selected only one physical activity measure and one cognitive measure for further testing with explanatory factors. We selected the measures with largest effect sizes in these initial sex-adjusted analyses.

#### Interactions and adjustments

Interactions between physical activity with APOE-ε4 and sex were tested to assess whether there was an effect modifying role of APOE-ε4 or sex (taking p<0.10 as evidence of an interaction). Multivariable linear regression modelling assessed the extent to which the association between physical activity and later-life cognition was attenuated by potentially explanatory factors including earlier-life covariables (childhood cognition, childhood SEP and educational attainment) (model 2); cardiovascular health (Framingham Risk Score) (model 3); emotional mental health (GHQ-28 derived case-level) (model 4) and APOE-ε4 status (model 5). Further adjustment was made for adulthood SEP and lifetime smoking status (details in online supplemental eFigure 2). Results are shown as mean difference in standardised cognitive scores with 95% CIs; adjustments were not made for multiple comparisons in line with previous studies.^35^

#### Physical activity with cognition: structural equation modelling approach

We additionally used an analytical life course approach to directly test how well different patterns of hypothesised exposure of physical activity fit to the real data. we compared an ‘accumulation’ model with a ‘sensitive period’ model. The ‘accumulation’ model proposes that the impact of participation is cumulative over the life course and that the longer an individual is active, the greater the impact on cognitive function at age 69. The ‘sensitive period’ model proposes that participation in physical activity during a particular stage in life (eg, time period 1, 2 or 3) has a greater effect on cognitive function at age 69 than activity outside this specified time period.

The structural equation modelling approach is outlined in greater detail in Mishra *et al* 36 and see online supplemental eTable 1, online supplemental eFigure 1. In brief, a pattern of physical activity exposure was created for each participant. To reduce multicollinearity of repeated measures, physical activity at every age was expressed in binary form as ‘not active’ (no participation in physical activity/month) and ‘active’ (participated one or more times/month); and the five physical activity assessments were condensed into three time periods: period 1 (ages 36 and 43), period 2 (age 53) and period 3 (ages 60 and 69), resulting in eight possible trajectories of physical activity exposure. F-tests were conducted to test how well hypothesised models of exposure fit the real data, compared with a reference model (fully saturated model). Hypothesised life course models included a model of no effect; an accumulation model; sensitive period 1; sensitive period 2 and sensitive period 3. Higher p values indicate that the life course model assessed fits the data as well as the reference model. The model with the lowest f value was deemed as the best fit.^25 36^ All analyses were sex adjusted.

We further assessed how these eight life course patterns of adulthood physical activity associated with cognitive state in linear regression models, adjusted for sex.

## Results

A flow chart of the analytical sample (max n=1531) is illustrated in figure 1. Table 1 shows sample characteristics (53% female). Generally, rates of physical inactivity increased with age, for example, rates of inactivity at age 36 were 33% rising to 56% at age 69.

**Table 1:** Descriptive characteristics of the analytical full sample

Max n	n (%)	1531 (100%)
Sex	Female (%)	53
Leisure time physical activity participation		
Physical activity at age 36	None (%)	33
	Moderate activity: 1–4 times/month (%)	27
	Most activity: ≥5 times/month (%)	40
Physical activity at age 43	None (%)	47
	Moderate activity: 1–4 times/month (%)	25
	Most activity: ≥5 times/month (%)	28
Physical activity at age 53	None (%)	42
	Moderate activity: 1–4 times/month (%)	21
	Most activity: ≥5 times/month (%)	37
Physical activity at ages 60–64	None (%)	61
	Moderate activity: 1–4 times/month (%)	15
	Most activity: ≥5 times/month (%)	24
Physical activity at age 69	None (%)	56
	Moderate activity: 1–4 times/month (%)	14
	Most activity: ≥5 times/month (%)	30
Physical activity, accumulative across five time periods	Never active	11
	Active at one time period (%)	17
	Active at two time periods (%)	20
	Active at three time periods (%)	20
	Active at four time periods (%)	17
	Active at all five time periods (%)	15
Cognitive function, age 69		
ACE-III total scores at age 69	Mean (SD)	92 (6.2)
Word learning test (WLT) score at age 69	Mean (SD)	22.5 (5.8)
Visual Search Speed score at age 69	Mean (SD)	261.6 (71.8)
Covariables		
Cognition score at age 8 (standardised*)	Mean (SD)	0.17 (7.8)
Childhood socioeconomic position	Manual (%)	51
	Non-manual (%)	49
Highest educational attainment up to age 26	None attempted (%)	28
	Vocational or GCSE (%)	29
	A-level or higher (%)	43
Framingham Risk Score at age 69	Median %, (IQR)	24.9 (14.0)
Mental health caseness at age 69	Yes (%)	13
APOE-ε4	APOE ε4 non-carriers (%)	70
	APOE ε4 carriers (%)	30

The analytical sample required complete physical activity data at 5 time periods and at least one available cognitive function measure at age 69.

*Standardised to the available sample at age 8 (n=4256).

ACE-III, Addenbrooke’s Cognitive Examination-III; GCSE, General Certificate of Secondary Education; NSHD, National Survey of Health and Development.

### Physical activity and later-life cognition

**Figure 2:**
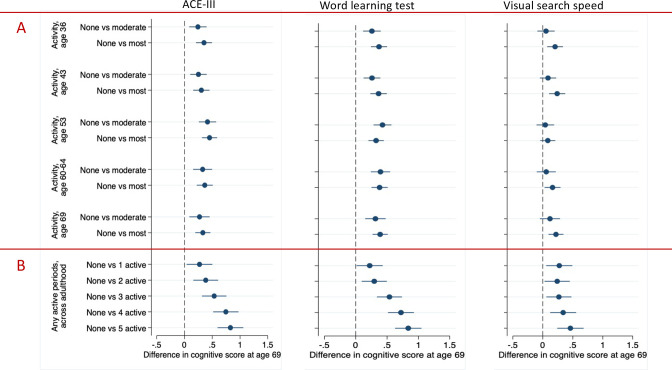
Associations between physical activity at assessed time periods (ages 36, 43, 53, 60 and 69), and cumulative physical activity across these time periods, with standardised cognitive function tests at age 69. Standardised coefficients (difference in SD of the cognitive test) and 95% CIs are presented from multiple linear regression sex-adjusted models, which compare cognitive test scores between: (A) those who were not active in a time period with those who were moderately active (participated in activity 1–4 times per month); and those who were most active (participated ≥5 times per month); (B) those who were never physically active in adulthood with those who were active (participated in activity ≥1 time per month) at varying durations across adulthood. ACE-III, Addenbrooke’s Cognitive Examination-III total test scores.

Compared with those not active, those who were moderately (1–4 activities per month) or most (≥5 activities per month) physically active, at any age, had significantly higher cognitive ACE-III and WLT scores at age 69 (illustrated in figure 2A and coefficients shown in online supplemental eTable 2). For ACE-III and WLT scores, the effects were broadly similar in those who were moderately and most active, and across all time periods.

For VSS scores, compared with those not active, those who were most physically active at any age had significantly higher cognitive VSS scores at age 69 (figure 2A). The association between moderate physical activity and VSS scores was less prominent.

For accumulative adulthood physical activity, compared with those who were never physically active across adulthood, those who were physically active (≥1 activity per month), to any extent in adulthood (≥1 time period), had higher cognitive scores at age 69 (figure 2B). There was evidence of an overall linear accumulation relationship (p<0.01), where being active across more time periods in adulthood was related to higher cognitive scores at age 69, particularly for ACE-III and WLT scores (figure 2).

The strongest association between any physical activity measure and any later-life cognitive measure (ie, the largest effect size) was between the accumulative adulthood physical activity and ACE-III scores at age 69; particularly for those who were active in five periods across adulthood (figure 2B and coefficients shown in online supplemental eTable 2); the accumulative physical activity measure and ACE-III scores were subsequently selected for further modelling.

### Interactions and adjustments

**Figure 3:**
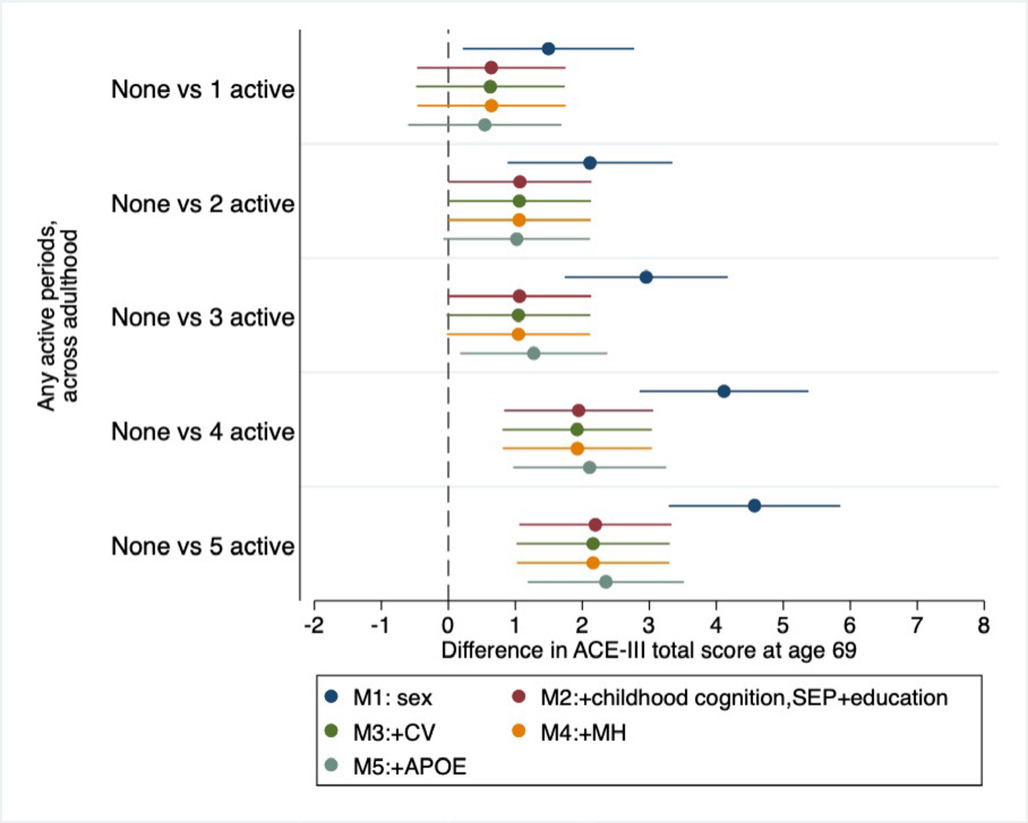
Associations between accumulative adulthood physical activity with Adenbrooke’s Cognitive Examination-III total score at age 69. Coefficients and 95% CIs are presented from multivariable linear regression models comparing those who were never physically active in adulthood with those who were active (participated in activity ≥1 times per month) at varying frequencies across adulthood. Models were adjusted for sex (blue); childhood cognition, childhood socioeconomic position (SEP) and education (red); cardiovascular (CV) health at age 69 (green); emotional mental health (MH) at age 69 (orange) and APOE-E4 status (grey).

Further investigation on the relationship between accumulative adulthood physical activity and ACE-III scores at age 69 revealed there was no significant APOE-ε4 (p=0.2) or sex (p=0.3) interactions. The inclusion of earlier-life variables, including childhood cognition, childhood SEP and educational attainment, attenuated the effect size between accumulative physical activity and cognitive ACE-III scores, by around a half to two-thirds in most cases, but results mainly remained significant at the 5% level (figure 3; M2). A stepwise adjustment of these earlier-life covariables is shown online supplemental eFigure 2 and demonstrates the additive attenuating influence of childhood cognition and educational attainment. Adjustment for cardiovascular risk at age 69 (figure 3; M3), mental health at age 69 (figure 3; M4) and APOE-ε4 status (figure 3; M5), own occupational attainment (online supplemental eFigure 2) and lifetime cigarette smoking status (online supplemental eFigure 2) did not further attenuate these associations. online supplemental eFigure 2 online supplemental eFigure 2

### Structured equation modelling

The structured equation modelling approach confirmed that for all three cognitive outcome measures an accumulation model provided a better fit for the data than any of the three sensitive period models, providing evidence that the impact of participation is cumulative over the life course and that the longer an individual is active, the greater the impact on cognitive function at age 69 (table 2).

**Table 2:** Comparing the relationship between different life course models of physical activity with cognitive function at age 69

Physical activity life course model	ACE-III, age 69		Word learning test, age 69		Visual search speed, age 69	
	f	P value	f	P value	f	P value
Saturated model	Reference		Reference		Reference	
No effect	12.42	<0.01	13.11	<0.01	2.61	<0.01
Accumulation	1.41	0.21	0.55	0.77	1.79	0.11
Sensitive period 1 (ages 36–43)	10.70	<0.01	10.05	<0.01	2.70	<0.01
Sensitive period 2 (age 53)	3.37	<0.01	6.31	<0.01	2.51	0.02
Sensitive period 3 (ages 60–69)	8.60	<0.01	6.92	<0.01	1.92	0.07
Best model*	**Accumulation**		**Accumulation**		**Accumulation**	

All models are adjusted for sex.

Categories at three age spans were chosen to represent physical activity in the life course and expressed in binary form (inactive vs moderately/most active). The ‘saturated model’ is the most complicated model that contains all parameters.

*Higher (less significant) p values indicate that the life course model assessed fits the data as well as the reference model; The lower the f value, the better the model fit.

ACE-III, Addenbrooke’s Cognitive Examination.

**Figure 4:**
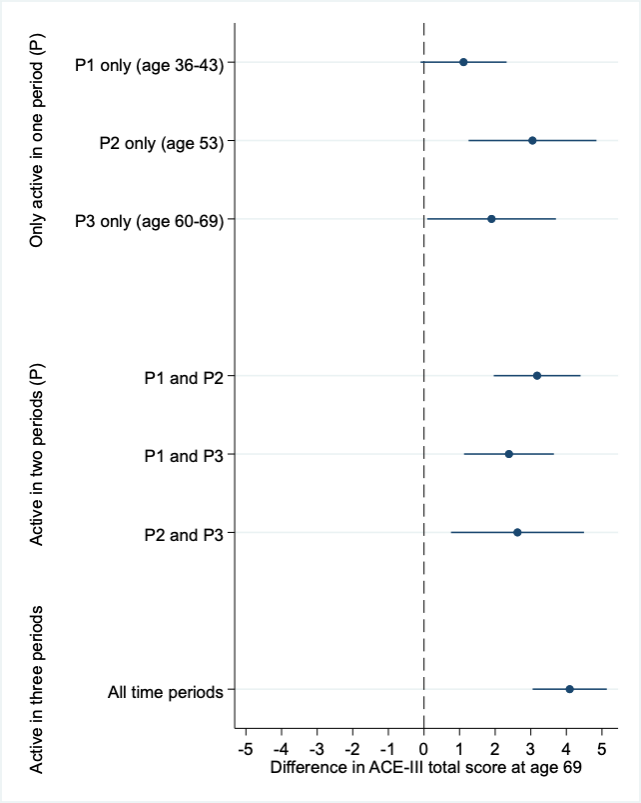
Associations between life course patterns of adulthood physical activity, compared with those never active, on the Adenbrooke’s Cognitive Examination (ACE-III) total score at age 69. coefficients (difference in the cognitive test) and 95% CIs are presented from multiple linear regression sex-adjusted models, which compare cognitive test scores between those who were never physically active in adulthood with those who were active (participated in activity ≥1 times per month) at varying periods across adulthood.

We further tested how physical activity patterns, based on their engagement across three time periods (period 1 earlier-life: ages 36 and 43; period 2 mid-life: age 53; period 3 later-life: ages 60 and 69), were associated with cognitive state. We found, compared with those who were never physically active, all patterns of physical activity engagement were associated with higher ACE-III scores at age 69, including those who were only active in earlier-life, mid-life or later-life (figure 4, online supplemental eFigure 1). However, this analysis also demonstrated that those who were active at all time periods had the strongest association with cognitive state.

## Discussion

We find compelling evidence of the effects of being, and remaining, physically active across 30 years of adulthood on later-life cognition in the 1946 British Birth cohort. Being physically active at all time points in adulthood was associated with higher cognitive performance and verbal memory scores at age 69. Notably, the effect sizes were similar across all adult ages, and for those who were either moderately or most physically active, suggesting that being physically active at any time in adulthood, even if participating as little as once per month, is linked with higher cognition. However, most effects were observed in those maintaining physical activity across adulthood. Finally, while the positive association between accumulative physical activity and later-life cognitive state was partly explained by childhood cognition, SEP and education, the effect remained significant when these were accounted for; and associations were not explained by differences in later-life cardiovascular health or mental health.

### Strengths and weaknesses

This study draws on the longest continuously running birth cohort, enabling a large age-homogeneous geographically representative sample of individuals born across mainland Britain. Other key strengths include the availability of multiple measures of physical activity collected prospectively over 30 years of adulthood, enabling us to uniquely assess timing of physical activity from a life course perspective with ‘sensitive’ periods or exercise maintenance effects; measures of a range of potential confounders including childhood cognition to reduce the plausibility of reverse directionality and reduce confounding bias; and performance on a range of cognitive domains in later-life, enabling us to demonstrate that cognitive state and memory domains were more sensitively linked to physical activity. Limitations are that there was a disproportional attrition of participants who were socially disadvantaged and less healthy,^37 38^ and that while representative of the UK population in 1946, the sample is exclusively white. Participants still involved at age 69 generally had higher childhood cognition and more education than the original cohort, so associations reported here may underestimate the strength of effects in those in the denominator population.^17 37^ Further, data on physical activity were only representing self-reported leisure-time activity and do not take into exercise adherence, exercise intensity or duration.^39^

### Comparison in relation to other studies

#### Timing

Previous studies have demonstrated links between physical activity in midlife or later-life with later-life cognition^5–7 15 16 39–41^ yet studies often have short follow-up periods, or are at risk of reverse directionality,^8^ limiting their ability to assess the role of timing or accumulation of physical activity. Work in this cohort previously demonstrated associations between midlife physical activity on midlife verbal memory^15^ and search speed^16^ decline. We expand on this by using physical activity measures spanning over 30 years of adulthood on a range of later-life cognitive domains, limiting the likelihood of reverse directionality. We demonstrated that being physically active, to a similar extent across all assessment periods, was linked with higher cognitive performance at age 69, suggesting there are not sensitive periods in which participating in physical activity has a greater effect on later-life cognitive function. Instead, our findings suggests that participating in physical activity, at any time in adulthood, is linked with higher later-life cognition. In addition, for cognitive state (ACE-III) and verbal memory scores (WLT), effect sizes were similar for those who were moderately (1–4 activities/month) and most (≥5 activities/month) physically active, suggesting that being physically active, to any extent (at least once/month), is associated with better cognitive health.

#### Accumulation

Few studies have directly assessed an accumulation effect of maintaining physical activity throughout the life course. We provide evidence, from the standard regression modelling and complementary structured equation modelling approaches, that the greatest cognitive effects were observed for those who maintained physical activity across adulthood in an accumulative dose-response manner. This suggests that lifelong maintenance of physical activity is the most optimal; the longer an individual is active, the more likely they are to have higher later-life cognitive function.

#### Explanatory factors

Unlike most studies, we were able to address the role of potential confounders and explanatory factors using data collected prospectively over nearly seven decades. We found that childhood cognition, childhood SEP and education explained around half to two-thirds of the relationship between cumulative physical activity and later-life cognition. We have previously demonstrated how these measures of advantage have independent effects on later-life cognitive state (ACE-III) in the cohort; childhood cognition showed the strongest effect, suggesting a prominent general ability component to later-life cognitive state that tracks back from childhood.^12^ More advantaged childhood socioeconomic background and educational attainment have been linked to enhanced physical activity in the cohort^12 42^ and may reflect the availability of, and a response to, opportunities to develop physical activity habits including practical skills, self-esteem and motivation, greater knowledge of exercise benefits, and financial resources for classes or clubs.^43 44^

However, and importantly, even after accounting for these explanatory factors, and limiting the likelihood of reverse directionality of those with higher cognitive ability and education more likely to engage in activity, we found there were still significant effects between accumulative physical activity and later-life cognition. This suggests there are additional pathways underlying this association. The relationship between accumulative physical activity and later-life cognition was not attenuated with adjustment of later-life cardiovascular or mental health, suggesting they are not important explanatory factors at this time.

### Meaning of the study

Together, these results suggest that the initiation and maintenance of physical activity across adulthood may be more important than the timing of participating in physical activity in the life course, or the frequency of physical activity at a specific period. Our findings support guidelines to recommend participation in any physical activity across adulthood and provide evidence that encouraging inactive adults to be more active at any time, and encouraging already active adults to maintain activity, could confer benefits on later-life cognition. The positive association between physical activity and later-life cognition support initiatives for better access to education which incorporates the development of practical skills and motivation for physical activity,^45^ as well as supporting initiatives to encourage engagement and maintenance of physical activity at all ages of the life span.

### Unanswered questions and future research

Our results warrant further investigation into the pathways underlying inferred benefits of physical activity on later-life cognition, including the hypothesised modulating role of activity in amyloid clearance, cerebral blood flow, inflammation and neurotrophic factors.^46^ Future research should seek to delineate the optimal beneficial aspects of physical activity interventions, such as the type, intensity, duration, adherence and setting, as well as exploring the role of key influences on physical activity, such as education, culture, life transitions and genetics. This work builds on existing and ongoing evidence in the cohort demonstrating engagement in physical activity can be embedded into a proxy of cognitive reserve.^47^ Continued follow-up will enable further investigation into whether physical activity confers beneficial effects on cognitive ageing, such as buffering cognitive deterioration in the presence of disease markers that cause dementia, and ultimately delaying dementia onset.

10.1136/jnnp-2022-329955.supp2 Supplementary data

## Data Availability

Data are available on reasonable request. Data used in this publication are available to bona fide researchers on request to the NSHD Data Sharing Committee via a standard application procedure. Further details can be found at http://www.nshd.mrc.ac.uk/data. doi:10.5522/NSHD/Q102; 10.5522/NSHD/Q103.
