# Supplementary material for: Timing of physical activity across adulthood on later-life cognition: 30 years follow-up in the 1946 British birth cohort
Source: J Neurol Neurosurg Psychiatry. 2023 Feb 21;94(5):349–56. doi: 10.1136/jnnp-2022-329955 (PMC10176405; doi:10.1136/jnnp-2022-329955)
Supplement: Supplementary data [file jnnp-2022-329955supp001.pdf]

Supplementary material

Supplementary eTable 1. Life course modelling distribution.

Period 1: Age 36-43	Period 2: Age 53	Period 3: Age 60-69	n	n %
0	0	0	165	11
1	0	0	235	15
0	1	0	60	4
0	0	1	58	4
1	1	0	218	14
1	0	1	185	12
0	1	1	56	4
1	1	1	554	36

To reduce multi-collinearity of repeated measures, categories at three age spans were chosen to represent physical activity in the life course and expressed in binary form (inactive vs moderately/most active), resulting in 8 possible trajectories.
Period 1: (ages 36-43)
Period 2: (age 53)
Period 3: (ages 60-69)

The 'saturated model' is the most complicated model that contains all parameters. The 'accumulation' model proposes that the impact of participation is cumulative over the life course and that the longer an individual is active, the greater the impact on cognitive function at age 69. A 'sensitive period' model proposes that participation in physical activity during a particular stage in life (e.g. time period 1, 2 or 3) has a greater effect on cognitive function at age 69 than activity outside this specified time period.

Fully saturated model:
$E(Y) = \alpha + \beta_1P_1 + \beta_2P_2 + \beta_3P_3$
$+ \theta_{12}P_1P_2 + \theta_{13}P_1P_3 + + \theta_{23}P_2P_3$
$+ \theta_{123}P_1P_2P_3$

Hypothesis and corresponding parameter constraints on regression analysis
Accumulation - effect size allowed to vary between time points
$\beta_1 \neq \beta_2 \neq \beta_3$
and
$\theta_{12}=\theta_{13}=\theta_{23}=\theta_{123}=0$
Sensitive period at 36+43
$\beta_2=\beta_3=\theta_{12}=\theta_{13}=\theta_{23}=\theta_{123}=0$
Sensitive period at 53
$\beta_1=\beta_3=\theta_{12}=\theta_{13}=\theta_{23}=\theta_{123}=0$
Sensitive period at 60-69
$\beta_1=\beta_2=\theta_{12}=\theta_{13}=\theta_{23}=\theta_{123}=0$

Supplementary eFigure 1: Associations between life course patterns of adulthood physical activity, compared to those never active, on the Adenbrooke's Cognitive Examination (ACE-III) total score at age 69.

Life course pattern

To reduce multi-collinearity of repeated measures, categories at three age spans were chosen to represent physical activity in the life course and expressed in binary form (inactive vs moderately/most active), resulting in 8 possible trajectories.

	Patterns of physical activity	n	%
0	Never active	165	11
1	Period 1 only (age 36-43)	235	15
2	Period 2 only (age 53)	60	4
3	Period 3 only (age 60-69)	58	4
4	Period 1 (age 36-43) +2 (age 53)	218	14
5	Period 1 (age 36-43) + 3 (age 60-69)	185	12
6	Period 2 (age 53) +3 (age 60-69)	56	4
7	Always active	554	36

Coefficients and 95% confidence intervals are presented from multivariable linear regression models comparing those who were never physically active in adulthood with those who were active (participated in activity ≥1 times per month) at varying time periods across adulthood.

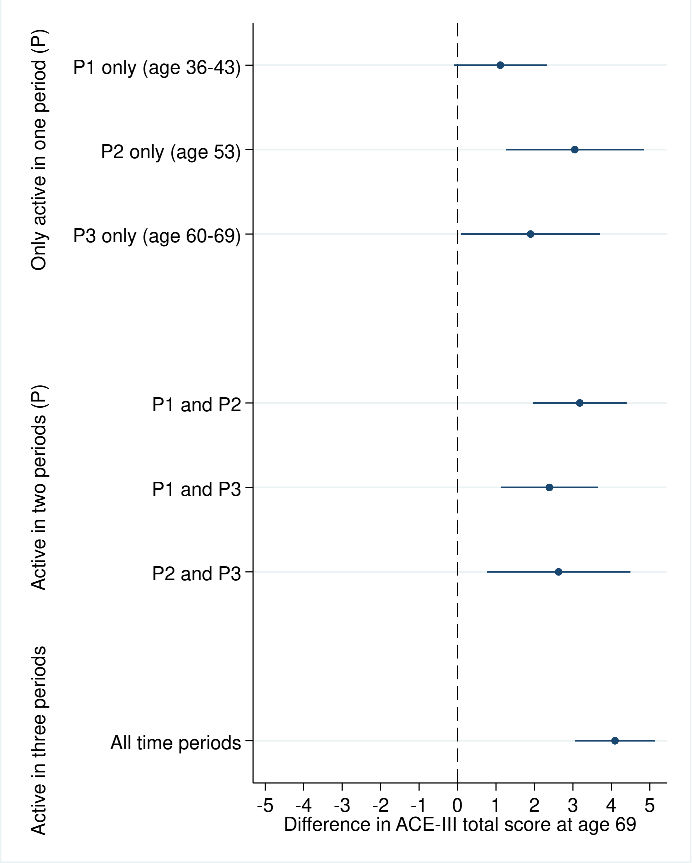

Supplementary eTable 2: Associations between physical activity at assessed time periods (ages 36, 43, 53, 60 and 69), and cumulative physical activity across these time periods, with standardised cognitive function tests at age 69.

		Standardised ACE-III at age 69			Standardised WLT at age 69			Standardised search speed at age 69		
		ß	p	95% C.I.	ß	p	95% C.I.	ß	p	95% C.I.
**A) Physical activity age 36**										
	No physical activity (reference)									
	Moderate physical activity: One to four times	0.24	**<0.01**	0.10-0.39	0.25	**<0.01**	0.11-0.38	0.03	0.67	-0.11-0.16
	Most physical activity: Five or more times	0.36	**<0.01**	0.22-0.49	0.35	**<0.01**	0.23-0.47	0.16	**0.01**	0.04-0.28
**Physical activity age 43**										
	No physical activity (reference)									
	Moderate physical activity: One to four times	0.25	**<0.01**	0.11-0.39	0.24	**<0.01**	0.11-0.36	0.05	0.4	-0.07-0.18
	Most physical activity: Five or more times	0.29	**<0.01**	0.15-0.42	0.35	**<0.01**	0.22-0.47	0.22	**<0.01**	0.10-0.35
**Physical activity age 53**										
	No physical activity (reference)									
	Moderate physical activity: One to four times	0.42	**<0.01**	0.27-0.56	0.4	**<0.01**	0.27-0.54	0.05	0.51	-0.09-0.18
	Most physical activity: Five or more times	0.48	**<0.01**	0.35-0.61	0.34	**<0.01**	0.22-0.45	0.13	**0.04**	0.01-0.24
**Physical activity age 60-64**										
	No physical activity (reference)									
	Moderate physical activity: One to four times	0.32	**<0.01**	0.16-0.48	0.37	**<0.01**	0.22-0.52	0.09	0.23	-0.06-0.24
	Most physical activity: Five or more times	0.4	**<0.01**	0.27-0.54	0.38	**<0.01**	0.26-0.50	0.18	**0.01**	0.05-0.30
**Physical activity age 69**										
	No physical activity (reference)									
	Moderate physical activity: One to four times	0.26	**<0.01**	0.09-0.43	0.33	**<0.01**	0.18-0.49	0.11	0.17	-0.05-0.27
	Most physical activity: Five or more times	0.31	**<0.01**	0.18-0.44	0.36	**<0.01**	0.24-0.47	0.22	**<0.01**	0.10-0.34
**B) Physical activity cumulative**										
	Never vs 1 period	0.26	**0.02**	0.05-0.48	0.24	**0.02**	0.05-0.44	0.22	**0.03**	0.02-0.42
	Never vs 2 periods	0.41	**<0.01**	0.20-0.61	0.3	**<0.02**	0.11-0.48	0.2	**0.05**	0.00-0.39
	Never vs 3 periods	0.54	**<0.01**	0.33-0.75	0.51	**<0.01**	0.32-0.70	0.25	**0.01**	0.06-0.45
	Never vs 4 periods	0.75	**<0.01**	0.54-0.96	0.73	**<0.00**	0.53-0.92	0.28	**0.01**	0.08-0.48
	Never vs 5 periods	0.84	**<0.01**	0.62-1.06	0.81	**<0.01**	0.62-1.01	0.44	**<0.01**	0.24-0.65

Standardised coefficients (difference in standard deviation of the cognitive test) and 95% confidence intervals are presented from multiple linear regression sex-adjusted models which compare cognitive test scores between: A) Those who were not active in a time period with those who were moderately active (participated in activity 1-4 times per month); and those who were most active (participated ≥5 times per month); B) Those who were never physically active in adulthood with those who were active (participated in activity ≥1 times per month) at varying periods across adulthood. ACE-III = Addenbrooke's Cognitive Examination-III total test scores.

Supplementary eFigure 2: Associations between cumulative adulthood physical activity with Adenbrooke's Cognitive Examination (ACE-III) total score at age 69.

Coefficients and 95% confidence intervals are presented from multivariable linear regression models comparing those who were never physically active in adulthood with those who were active (participated in activity ≥1 times per month) at varying frequencies across adulthood. Models were adjusted for sex (blue); childhood cognition (red); childhood socioeconomic position (cSEP; green); education (orange); adulthood socioeconomic position (aSEP; grey); lifetime cigarette use up to age 69 (pink).

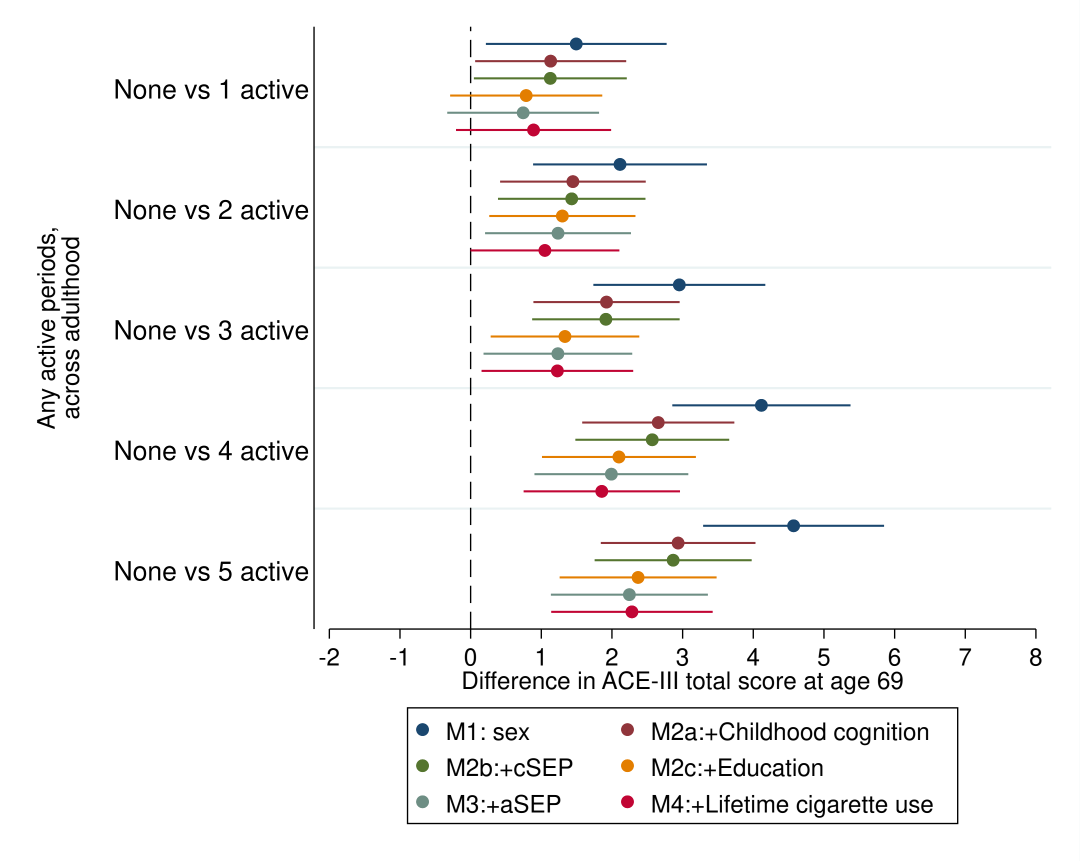

<u>Childhood cognition:</u> The standardized sum of four tests of verbal and nonverbal ability at age 8 represented childhood cognition.

<u>Childhood SEP:</u> Recorded from paternal occupation according to the Registrar General's classification of the paternal occupation [29] and dichotomized into 'Unskilled, partly skilled, or manual skilled' and 'Non-manual skilled, intermediate or professional'.

<u>Education:</u> Recorded up to age 26 was categorized into three groups based on the Burnham Scale [30]: 'None attempted'; 'Vocational or ordinary (O' level or equivalent)'; 'advanced (A-Level) or higher education'.

<u>Adulthood SEP:</u> Recorded from occupation up to age 60 according to the Registrar General's classification of the paternal occupation [29] and dichotomized into 'Unskilled, partly skilled, or manual skilled' and 'Non-manual skilled, intermediate or professional'.

<u>Lifetime cigarette use:</u> Assessed up to age 69 and operationalised into i) never smoker; ii) former smoker; current smoker.

Supplementary eTable 3: Descriptive characteristics of the complete case sample.

		Complete case sample
max n	n (%)	1237 (100%)
Sex	Female (%)	659 (53%)
Leisure time physical activity participation		
Physical activity at age 36	None (%)	396 (32%)
	Moderate activity: 1-4 times/month (%)	341 (27%)
	Most activity: ≥5 times/month (%)	500 (40%)
Physical activity at age 43	None (%)	587 (47%)
	Moderate activity: 1-4 times/month (%)	315 (25%)
	Most activity: ≥5 times/month (%)	335 (27%)
Physical activity at age 53	None (%)	512 (42%)
	Moderate activity: 1-4 times/month (%)	473 (22%)
	Most activity: ≥5 times/month (%)	452 (36%)
Physical activity at age 60-64	None (%)	743 (60%)
	Moderate activity: 1-4 times/month (%)	190 (15%)
	Most activity: ≥5 times/month (%)	304 (25%)
Physical activity at age 69	None (%)	690 (55%)
	Moderate activity: 1-4 times/month (%)	168 (14%)
	Most activity: ≥5 times/month (%)	379 (31%)
Physical activity, accumulative across 5 time periods	Never active	132 (11%)
	Active at 1 time period (%)	204 (17%)
	Active at 2 time periods (%)	246 (20%)
	Active at 3 time periods (%)	251 (20%)
	Active at 4 time periods (%)	212 (17%)
	Active at all 5 time periods (%)	192 (15%)
Cognitive function, age 69		
ACE-III total scores at age 69	Mean (SD)	92 (5.5)
Visual search speed score at age 69	Mean (SD)	263.0 (71.8)
Word learning test score at age 69	Mean (SD)	22.4 (6.0)
Covariables		
Cognition score at age 8 (standardised*)	Mean (SD)	0.17 (0.8)
Childhood socioeconomic position	Manual (%)	644 (52%)
	Non-manual (%)	598 (48%)
Highest educational attainment up to age 26	None attempted (%)	339 (27%)
	Vocational or GCSE (%)	368 (30%)
	A-Level or higher (%)	530 (43%)
Framingham Risk Score at age 69	Median %, (IQR)	22.5 (14-33)
Mental health caseness at age 69	Yes (%)	236 (19%)
APOE-ε4	APOE ε4 non-carriers (%)	805 (70%)
	APOE ε4 carriers (%)	339 (30%)

NSHD, National Survey of Health and Development. SD, standard deviation. APOE: apolipoprotein E. ACE-III, Addenbrooke's Cognitive Examination-III. GCSE: General Certificate of Secondary Education. FHS-CVS: Framingham Heart Study-cardiovascular risk score; IQR=interquartile range.
**standardised to the available sample at age 8 (n=4256).*
The analytical sample required complete physical activity data at 5 time periods and at least one available cognitive function measure at age 69.

Supplementary eFigure 2: Associations between physical activity at assessed time periods (ages 36, 43, 53, 60 and 69), and cumulative physical activity across these time periods, with standardised cognitive function tests at age 69 in the complete-case sample (max n=1237).

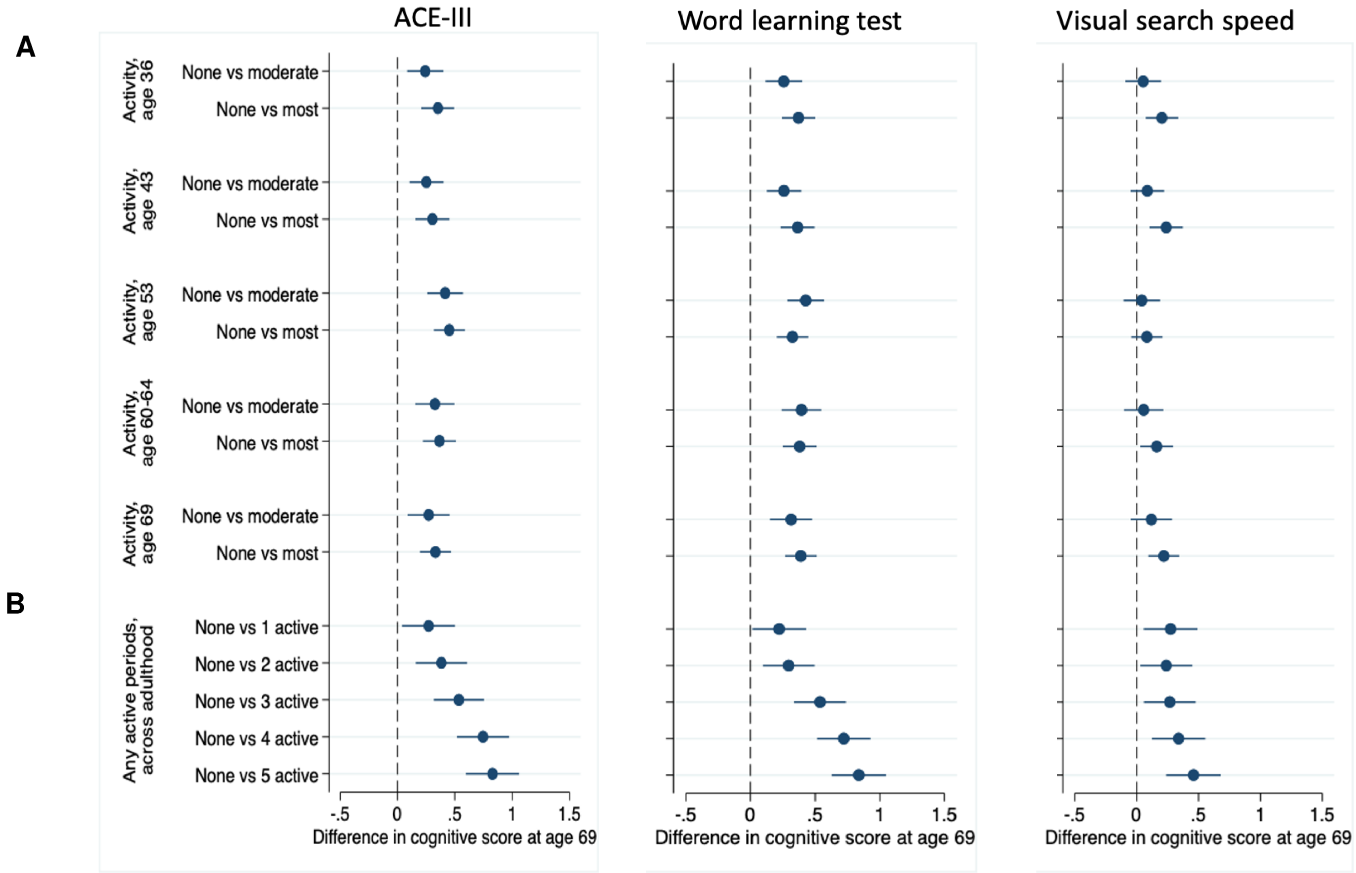

Standardised coefficients and 95% confidence intervals are presented from multiple linear regression sex-adjusted models which compare cognitive test scores between:
A) Those who were not active in a time period with those who were moderately active (participated in activity 1-4 times per month); and those who were most active (participated ≥5 times per month);
B) Those who were never physically active in adulthood with those who were active (participated in activity ≥1 times per month) at varying periods across adulthood. ACE-III = Addenbrooke's Cognitive Examination-III total test scores.

Supplementary eFigure 3: Associations between accumulative adulthood physical activity with Adenbrooke's Cognitive Examination (ACE-III) total score at age 69 in the complete-case sample (max n=1237).

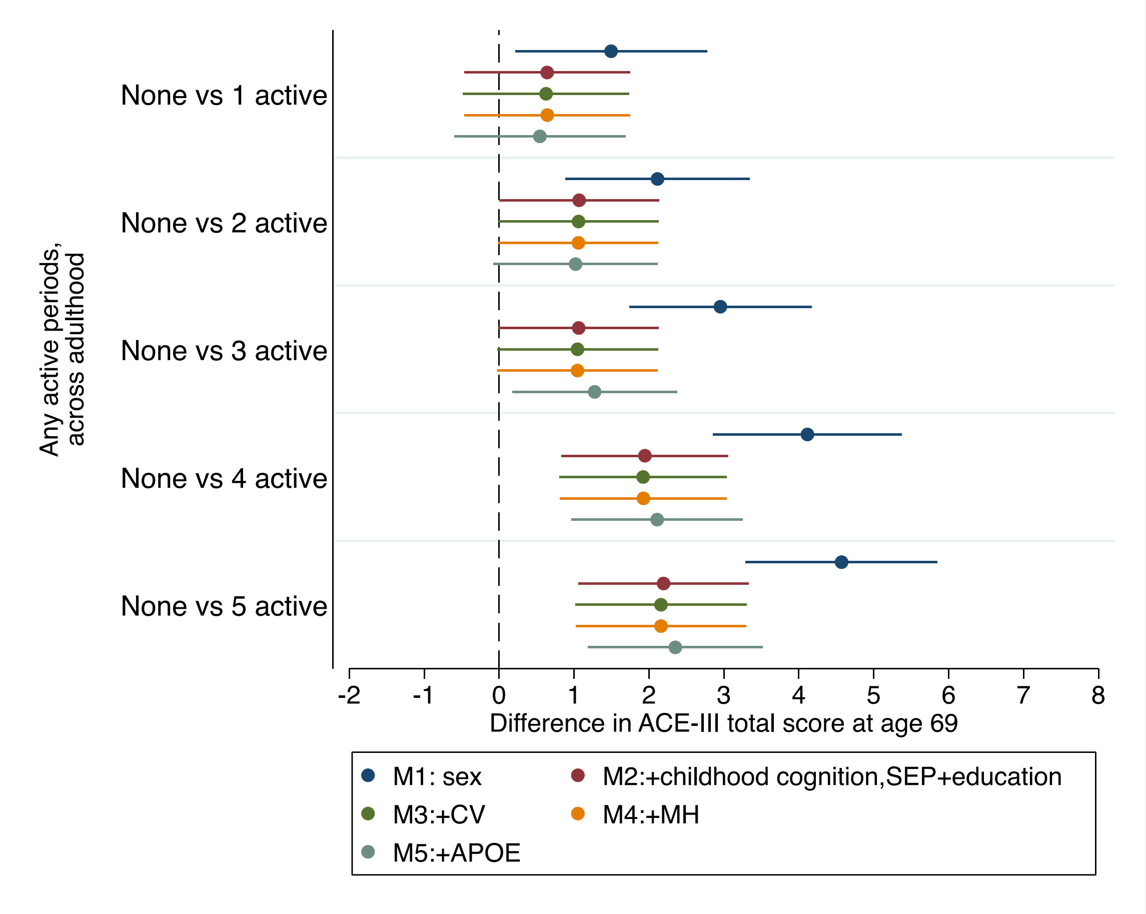

Coefficients and 95% confidence intervals are presented from multivariable linear regression models comparing those who were never physically active in adulthood with those who were active (participated in activity ≥1 times per month) at varying frequencies across adulthood. Models were adjusted for sex (blue); childhood cognition, social economic position (SEP) and education (red); cardiovascular health at age 69 (CV) (green); emotional mental health (MH) at age 69 (orange); and APOE-E4 status (grey).
