# Supplementary material for: Timing of physical activity across adulthood on later-life cognition: 30 years follow-up in the 1946 British birth cohort
Source: J Neurol Neurosurg Psychiatry. 2023 Feb 21;94(5):349–56. doi: 10.1136/jnnp-2022-329955 (PMC10176405; doi:10.1136/jnnp-2022-329955)
Supplement: Supplementary data [file jnnp-2022-329955supp002.pdf]

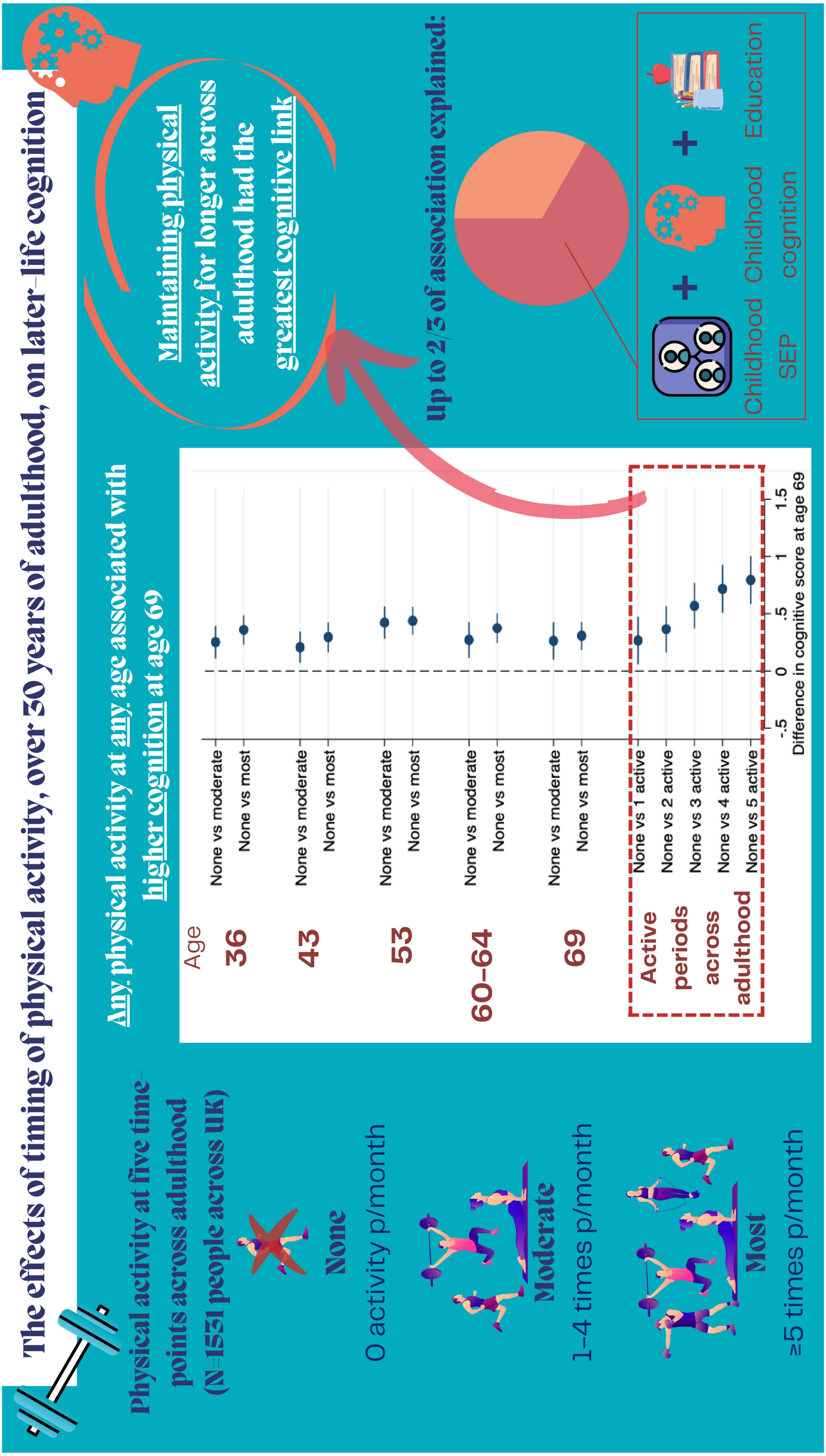
The effects of timing of physical activity, over 30 years of adulthood, on later-life cognition
Physical activity at five time-points across adulthood (N=1531 people across UK)
None
0 activity p/month
Moderate
1–4 times p/month
Most
≥5 times p/month
Any physical activity at any age associated with higher cognition at age 69
Age
36
None vs moderate
None vs most
43
None vs moderate
None vs most
53
None vs moderate
None vs most
60–64
None vs moderate
None vs most
69
None vs moderate
None vs most
Active periods across adulthood
None vs 1 active
None vs 2 active
None vs 3 active
None vs 4 active
None vs 5 active
-.5
0
.5
1
1.5
Difference in cognitive score at age 69
Maintaining physical activity for longer across adulthood had the greatest cognitive link
Up to 2/3 of association explained:
Childhood SEP
+
Childhood cognition
+
Education
